# Tetra­kis(di­cyclo­hexyl­amido)­zirconium(IV)

**DOI:** 10.1107/S2414314620011451

**Published:** 2020-09-04

**Authors:** Nils Frerichs, Marc Schmidtmann, Rüdiger Beckhaus

**Affiliations:** a Carl von Ossietzky Universität Oldenburg, Fakultät V, Institut für Chemie, Carl-von-Ossietzky-Strasse 9-11, D-26129 Oldenburg, Germany; Vienna University of Technology, Austria

**Keywords:** crystal structure, zirconium, amide, isotypism

## Abstract

The reaction of ZrCl_4_ with three equivalents of LiNCy_2_ (Cy is cyclo­hex­yl) resulted in the formation of tris­(di­cyclo­hexyl­amido)­zirconium chloride and the title compound, [Zr(C_12_H_22_N)_4_]. The latter is isotypic with its cerium(IV) analogue and crystallizes with three independent mol­ecules in the asymmetric unit. One mol­ecule is located about a twofold rotation axis, and the other two on fourfold inversion axes. In each mol­ecule, the Zr^IV^ atom has a distorted tetra­hedral coordination environment. The crystal under investigation was twinned by inversion in a 1:1 ratio.

## Abstract 2

The crystal structure of Zr(C_12_H_22_N)_4_ is isotypic with its cerium(IV) analogue.

## Graphical abstract

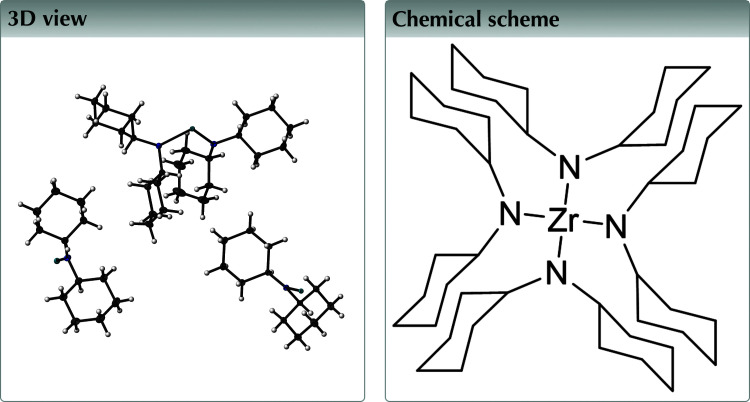

## Structure description

Amido complexes of group 4 metals play an important role in synthetic chemistry. They are widely used as catalysts in hydro­amino­alkyl­ation reactions (Roesky *et al.*, 2009 ▸) or in the catalysis of olefin polymerization reactions (Shafir & Arnold, 2001 ▸; Motolko *et al.*, 2017 ▸). Tetra­kis(di­alkyl­amido)­zirconium(IV) compounds are commonly known as precursors of a variety of more complex zirconium-containing compounds (Diamond *et al.*, 1995 ▸, 1996 ▸). Amido ligands are known for their ability to stabilize electron-deficient transition-metal complexes by N(*p*_π_)—*M*(*d*_π_) inter­actions (Yu *et al.*, 2004 ▸). Combined with the possibility of double substitution at the nitro­gen atom, which allows a broad variety in ligand design (Kasani *et al.*, 1997 ▸), amido ligands appear to be an inter­esting alternative to Cp-based ligands (Kempe, 2000 ▸; Guérin *et al.*, 2000 ▸). In particular, di­cyclo­hexyl­amine seems to be useful due to its steric demand that is similar to cyclo­penta­dienyl ligands (Duan *et al.*, 1997 ▸). Additionally, di­cyclo­hexyl­amido complexes of group 4 metals show close contacts between the central metal cation and the carbon atom of the CH group of one di­cyclo­hexyl­amido ligand, which is an indicator for attractive agostic inter­actions (Duan *et al.*, 1997 ▸; Adler *et al.*, 2014*b* ▸). The understanding of these inter­actions is very important (Scherer *et al.*, 2010 ▸) because they are considered to be inter­mediates in C—H bond-activation processes.

**Figure 1:**
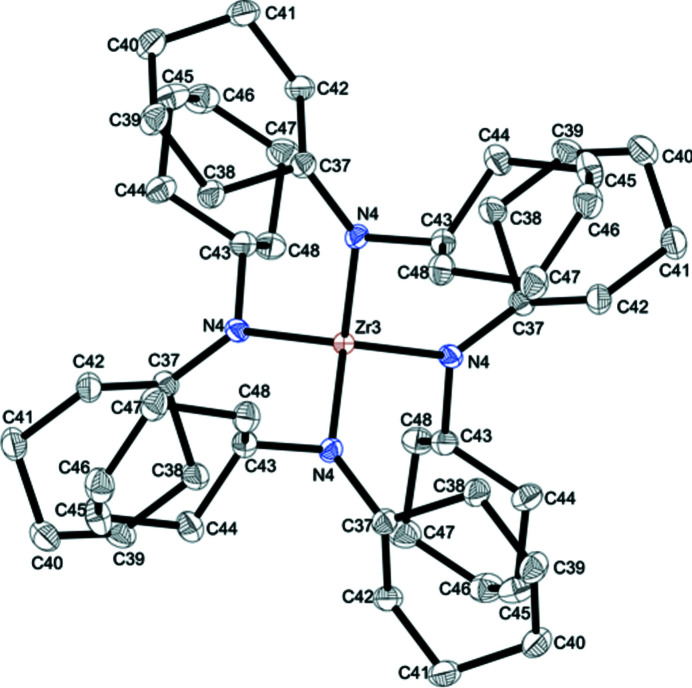
Representative for the three different mol­ecules of **1** in the asymmetric unit, the mol­ecular structure of complex Zr3 is displayed. Displacement ellipsoids correspond to the 50% probability level. H atoms have been omitted for clarity.

The title compound, **1**, crystallizes in the tetra­gonal space group *P* 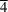 and is isostructural with tetra­kis­(di­cyclo­hexyl­amido)­cerium(IV) (Hitchcock *et al.*, 2006 ▸). The structure of **1** exhibits three independent mol­ecules, two of which lie on a fourfold inversion axis (Zr2, Zr3) as well as one (Zr1) lying on a twofold rotation axis. One of these mol­ecules is shown in Fig. 1 ▸. In each mol­ecule, the zirconium(IV) atom is coordinated in a slightly distorted tetra­hedral fashion with bond angles around zirconium(IV) ranging from 104.53 (8) to 112.00 (4)°. The nitro­gen atoms have a trigonal planar environment (sum of angles: N1: 359.2°, N2: 359.9°, N3: 359.6°, N4: 359.6°). The mean Zr—N bond length of 2.094 Å is slightly elongated compared to tris­(di­cyclo­hexyl­amido)­zirconium(IV) chloride (mean 2.044 Å; Adler *et al.*, 2014*a* ▸) but still short for a Zr—N single bond, indicating N(*p*_π_)—Zr(*d*_π_) inter­actions (Pyykkö & Atsumi *et al.*, 2009 ▸). The slight elongation can either be caused by a greater steric hindrance or by a less elctrophilic central metal atom in complex **1**. The large Zr—N—C bond angles [*e.g.* Zr3—N4—C37 = 138.00 (10)°, Zr3—N4—C43 = 108.15 (9)°] indicate that agostic inter­actions are not present.

**Figure 2:**
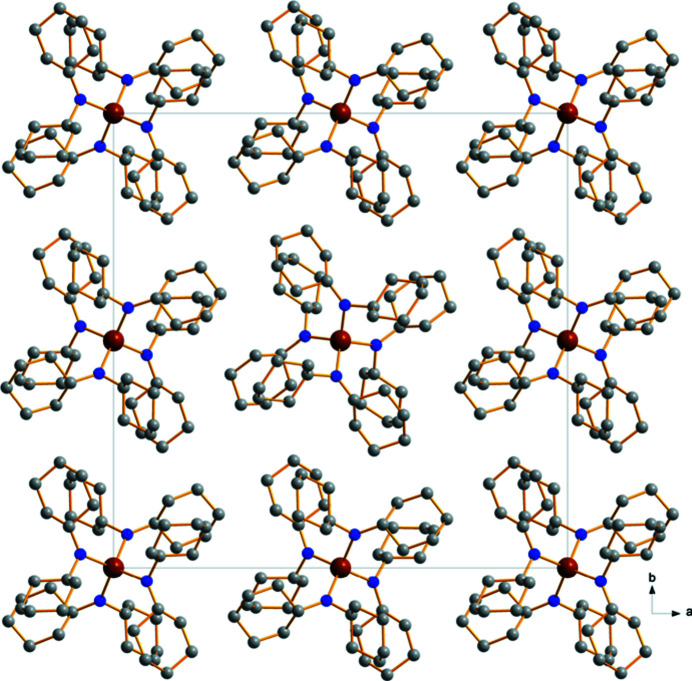
A view along the *c* axis showing the packing of individual mol­ecules in the crystal of **1**. No significant supra­molecular features can be observed. Colour code: C grey, N blue, Zr brown spheres.

No significant supra­molecular features are observed in the crystal structure of **1**. The molecular packing (Fig. 2 ▸) appears to be dominated by van der Waals inter­actions only.

## Synthesis and crystallization

All reactions were carried out under a dry nitro­gen atmosphere using Schlenk techniques or in a glove box. Lithium di­cyclo­hexyl­amide was synthesized by treatment of di­cyclo­hexyl­amine with one equivalent of *n*-butyl­lithium (2.5 *M* in *n*-hexa­ne). Solvents were dried according to standard procedures over Na/K alloy with benzo­phenone as indicator and distilled under a nitro­gen atmosphere.

Zirconiumtetra­chloride and three equivalents of lithium di­cyclo­hexyl­amide were suspended in 50 ml of *n*-hexane. After 16 h the reaction mixture was filtered hot through a P4-frit and stored at 243 K overnight. The solvent was deca­nted. After renewed storage of the mother liquor at 243 K, the title compound **1** crystallized in form of colourless blocks as the minor product, besides the mainproduct tris­(di­cyclo­hexyl­amido)­zirconium(IV) chloride.

## Refinement

Crystal data, data collection and structure refinement details are summarized in Table 1 ▸. The crystal under investigation was twinned by inversion in a 1:1 ratio.

## Supplementary Material

Crystal structure: contains datablock(s) I. DOI: 10.1107/S2414314620011451/wm5575sup1.cif

Structure factors: contains datablock(s) I. DOI: 10.1107/S2414314620011451/wm5575Isup3.hkl

CCDC reference: 2024547

Additional supporting information:  crystallographic information; 3D view; checkCIF report

## Figures and Tables

**Table 1:** Experimental details

Crystal data	
Chemical formula	[Zr(C_12_H_22_N)_4_]
*M*_r_	812.44
Crystal system, space group	Tetragonal, *P* 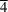
Temperature (K)	100
*a*, *c* (Å)	21.0321 (7), 10.2412 (5)
*V* (Å^3^)	4530.2 (4)
*Z*	4
Radiation type	Mo *K*α
μ (mm^−1^)	0.28
Crystal size (mm)	0.15 × 0.11 × 0.07

Data collection	
Diffractometer	Bruker Photon III CPAD
Absorption correction	Multi-scan (*SADABS*; Krause *et al.*, 2015 ▸)
*T*_min_, *T*_max_	0.936, 1.000
No. of measured, independent and observed [*I* > 2σ(*I*)] reflections	350034, 21937, 20806
*R*_int_	0.047
(sin θ/λ)_max_ (Å^−1^)	0.833

Refinement	
*R*[*F* ^2^ > 2σ(*F* ^2^)], *wR*(*F* ^2^), *S*	0.030, 0.080, 1.07
No. of reflections	21937
No. of parameters	479
H-atom treatment	H-atom parameters constrained
Δρ_max_, Δρ_min_ (e Å^−3^)	2.50, −0.42
Absolute structure	Refined as an inversion twin.
Absolute structure parameter	0.491 (17)

Computer programs: *APEX3* and *SAINT* (Bruker, 2015 ▸), *SHELXS* (Sheldrick, 2008 ▸), *SHELXL* (Sheldrick, 2015 ▸), *DIAMOND* (Brandenburg, 2006 ▸) and *publCIF* (Westrip, 2010 ▸).

## full crystallographic data

### Crystal data

**Table:**

[Zr(C_12_H_22_N)_4_]	*D*_x_ = 1.191 Mg m^−^^3^
*M**_r_* = 812.44	Mo *K*α radiation, λ = 0.71073 Å
Tetragonal, *P*4	Cell parameters from 9855 reflections
*a* = 21.0321 (7) Å	θ = 2.2–36.3°
*c* = 10.2412 (5) Å	µ = 0.28 mm^−^^1^
*V* = 4530.2 (4) Å^3^	*T* = 100 K
*Z* = 4	Block, colourless
*F*(000) = 1776	0.15 × 0.11 × 0.07 mm

### Data collection

**Table:**

Bruker Photon III CPAD diffractometer	20806 reflections with *I* > 2σ(*I*)
Radiation source: IµS microfocus	*R*_int_ = 0.047
φ and ω scans	θ_max_ = 36.3°, θ_min_ = 1.4°
Absorption correction: multi-scan (*SADABS*; Krause *et al.*, 2015)	*h* = −34→35
*T*_min_ = 0.936, *T*_max_ = 1.000	*k* = −35→35
350034 measured reflections	*l* = −17→17
21937 independent reflections	

### Refinement

**Table:**

Refinement on *F*^2^	Secondary atom site location: difference Fourier map
Least-squares matrix: full	Hydrogen site location: difference Fourier map
*R*[*F*^2^ > 2σ(*F*^2^)] = 0.030	H-atom parameters constrained
*wR*(*F*^2^) = 0.080	*w* = 1/[σ^2^(*F*_o_^2^) + (0.045*P*)^2^ + 0.9*P*] where *P* = (*F*_o_^2^ + 2*F*_c_^2^)/3
*S* = 1.07	(Δ/σ)_max_ < 0.001
21937 reflections	Δρ_max_ = 2.50 e Å^−^^3^
479 parameters	Δρ_min_ = −0.42 e Å^−^^3^
0 restraints	Absolute structure: Refined as an inversion twin.
Primary atom site location: structure-invariant direct methods	Absolute structure parameter: 0.491 (17)

### Special details

**Table:**

Geometry. All esds (except the esd in the dihedral angle between two l.s. planes) are estimated using the full covariance matrix. The cell esds are taken into account individually in the estimation of esds in distances, angles and torsion angles; correlations between esds in cell parameters are only used when they are defined by crystal symmetry. An approximate (isotropic) treatment of cell esds is used for estimating esds involving l.s. planes.
Refinement. Refined as a 2-component inversion twin.

### Fractional atomic coordinates and isotropic or equivalent isotropic displacement parameters (Å^2^)

**Table:**

	*x*	*y*	*z*	*U*_iso_*/*U*_eq_	
Zr1	0.500000	0.000000	0.15631 (2)	0.00900 (3)	
N1	0.42803 (6)	0.03193 (6)	0.03168 (12)	0.01353 (19)	
N2	0.53112 (6)	0.07285 (6)	0.27997 (12)	0.01322 (19)	
C1	0.39758 (6)	−0.02236 (7)	−0.03612 (14)	0.0134 (2)	
H1	0.413064	−0.061668	0.008681	0.016*	
C2	0.41569 (7)	−0.02952 (7)	−0.18129 (15)	0.0163 (2)	
H2A	0.401743	0.008770	−0.229671	0.020*	
H2B	0.462503	−0.032626	−0.189091	0.020*	
C3	0.38522 (7)	−0.08849 (8)	−0.24255 (17)	0.0195 (3)	
H3A	0.396690	−0.090539	−0.336209	0.023*	
H3B	0.402176	−0.127059	−0.199467	0.023*	
C4	0.31327 (8)	−0.08745 (9)	−0.22921 (17)	0.0223 (3)	
H4A	0.295261	−0.127056	−0.266107	0.027*	
H4B	0.295784	−0.051109	−0.279063	0.027*	
C5	0.29407 (8)	−0.08151 (9)	−0.08555 (17)	0.0217 (3)	
H5A	0.247231	−0.078131	−0.078933	0.026*	
H5B	0.307581	−0.120080	−0.037598	0.026*	
C6	0.32481 (7)	−0.02284 (8)	−0.02371 (16)	0.0180 (2)	
H6A	0.313233	−0.021210	0.069932	0.022*	
H6B	0.307492	0.015779	−0.066020	0.022*	
C7	0.40759 (7)	0.09482 (7)	−0.01821 (15)	0.0164 (2)	
H7	0.387575	0.087786	−0.105595	0.020*	
C8	0.35773 (8)	0.12664 (8)	0.06910 (16)	0.0203 (3)	
H8A	0.319517	0.099238	0.073568	0.024*	
H8B	0.374995	0.130846	0.158614	0.024*	
C9	0.33865 (11)	0.19220 (10)	0.0188 (2)	0.0315 (4)	
H9A	0.308303	0.211950	0.080587	0.038*	
H9B	0.317119	0.187739	−0.066653	0.038*	
C10	0.39710 (13)	0.23528 (10)	0.0037 (2)	0.0360 (5)	
H10A	0.383906	0.276766	−0.033078	0.043*	
H10B	0.416321	0.243068	0.090488	0.043*	
C11	0.44601 (12)	0.20469 (9)	−0.0856 (2)	0.0321 (4)	
H11A	0.484383	0.231896	−0.089201	0.039*	
H11B	0.428358	0.201710	−0.174997	0.039*	
C12	0.46478 (8)	0.13813 (7)	−0.03852 (17)	0.0205 (3)	
H12A	0.493600	0.118537	−0.103513	0.025*	
H12B	0.488376	0.141923	0.044763	0.025*	
C13	0.47597 (7)	0.10288 (7)	0.34634 (14)	0.0134 (2)	
H13	0.437144	0.086612	0.300953	0.016*	
C14	0.47425 (7)	0.17561 (7)	0.33277 (15)	0.0163 (2)	
H14A	0.476775	0.186916	0.239075	0.020*	
H14B	0.511975	0.193844	0.376808	0.020*	
C15	0.41395 (8)	0.20536 (8)	0.39145 (17)	0.0187 (3)	
H15A	0.416967	0.252264	0.386074	0.022*	
H15B	0.376491	0.191799	0.339954	0.022*	
C16	0.40488 (8)	0.18573 (8)	0.53359 (17)	0.0206 (3)	
H16A	0.438860	0.204958	0.587585	0.025*	
H16B	0.363511	0.201918	0.565547	0.025*	
C17	0.40676 (8)	0.11366 (7)	0.54837 (17)	0.0190 (3)	
H17A	0.369648	0.094844	0.503191	0.023*	
H17B	0.403796	0.102488	0.642077	0.023*	
C18	0.46806 (7)	0.08557 (7)	0.49133 (15)	0.0163 (2)	
H18A	0.467168	0.038726	0.500637	0.020*	
H18B	0.505004	0.101798	0.541109	0.020*	
C19	0.59346 (7)	0.09533 (7)	0.32831 (15)	0.0140 (2)	
H19	0.585789	0.118139	0.412521	0.017*	
C20	0.62529 (7)	0.14248 (7)	0.23477 (16)	0.0165 (2)	
H20A	0.629973	0.122311	0.147959	0.020*	
H20B	0.597560	0.180189	0.224499	0.020*	
C21	0.69080 (8)	0.16400 (8)	0.28315 (17)	0.0199 (3)	
H21A	0.710560	0.191981	0.216860	0.024*	
H21B	0.685718	0.188867	0.364562	0.024*	
C22	0.73425 (7)	0.10744 (8)	0.30895 (18)	0.0201 (3)	
H22A	0.775288	0.122574	0.344652	0.024*	
H22B	0.742873	0.084843	0.226011	0.024*	
C23	0.70308 (7)	0.06202 (8)	0.40578 (16)	0.0190 (3)	
H23A	0.697908	0.083749	0.490876	0.023*	
H23B	0.731086	0.024760	0.419236	0.023*	
C24	0.63806 (7)	0.03938 (7)	0.35741 (16)	0.0165 (2)	
H24A	0.618450	0.011776	0.424551	0.020*	
H24B	0.643759	0.013773	0.277151	0.020*	
Zr2	0.000000	0.000000	0.500000	0.00830 (5)	
N3	0.02989 (5)	0.07316 (5)	0.37512 (13)	0.01094 (19)	
C25	−0.02635 (6)	0.10124 (6)	0.30879 (14)	0.01102 (19)	
H25	−0.064700	0.083485	0.353538	0.013*	
C26	−0.03299 (7)	0.08426 (6)	0.16306 (13)	0.0131 (2)	
H26A	0.004274	0.100798	0.114754	0.016*	
H26B	−0.033604	0.037445	0.153159	0.016*	
C27	−0.09397 (8)	0.11227 (7)	0.10426 (16)	0.0173 (2)	
H27A	−0.096220	0.101243	0.010391	0.021*	
H27B	−0.131371	0.093295	0.148158	0.021*	
C28	−0.09609 (8)	0.18446 (7)	0.11954 (17)	0.0193 (3)	
H28A	−0.061017	0.203850	0.068839	0.023*	
H28B	−0.136779	0.200844	0.084477	0.023*	
C29	−0.08982 (8)	0.20312 (7)	0.26268 (16)	0.0170 (2)	
H29A	−0.127795	0.188354	0.311098	0.020*	
H29B	−0.087898	0.250028	0.269813	0.020*	
C30	−0.02993 (7)	0.17417 (6)	0.32438 (15)	0.0136 (2)	
H30A	−0.029115	0.184846	0.418515	0.016*	
H30B	0.008068	0.193517	0.283440	0.016*	
C31	0.09123 (6)	0.09799 (6)	0.32725 (14)	0.0122 (2)	
H31	0.082512	0.120210	0.242805	0.015*	
C32	0.12070 (7)	0.14711 (7)	0.41949 (15)	0.0145 (2)	
H32A	0.126836	0.127523	0.506477	0.017*	
H32B	0.090924	0.183228	0.429740	0.017*	
C33	0.18483 (7)	0.17221 (7)	0.36974 (18)	0.0187 (3)	
H33A	0.178124	0.196300	0.287836	0.022*	
H33B	0.203137	0.201631	0.435169	0.022*	
C34	0.23144 (7)	0.11790 (8)	0.34465 (16)	0.0188 (3)	
H34A	0.271448	0.135068	0.307915	0.023*	
H34B	0.241576	0.096291	0.427972	0.023*	
C35	0.20235 (7)	0.07030 (8)	0.24929 (16)	0.0180 (3)	
H35A	0.232214	0.034445	0.236673	0.022*	
H35B	0.196087	0.091219	0.163647	0.022*	
C36	0.13850 (7)	0.04449 (7)	0.29786 (15)	0.0150 (2)	
H36A	0.120230	0.015980	0.230724	0.018*	
H36B	0.145525	0.019146	0.378032	0.018*	
Zr3	0.500000	0.500000	0.500000	0.01251 (5)	
N4	0.49034 (6)	0.42185 (6)	0.62514 (14)	0.0149 (2)	
C37	0.52996 (7)	0.36924 (7)	0.67418 (16)	0.0166 (2)	
H37	0.509870	0.353480	0.756545	0.020*	
C38	0.59725 (8)	0.39077 (8)	0.70820 (17)	0.0202 (3)	
H38A	0.595089	0.423826	0.776885	0.024*	
H38B	0.617112	0.410036	0.629997	0.024*	
C39	0.63894 (8)	0.33570 (9)	0.75637 (18)	0.0236 (3)	
H39A	0.682655	0.351483	0.771725	0.028*	
H39B	0.622067	0.319897	0.840579	0.028*	
C40	0.64117 (9)	0.28090 (9)	0.65863 (18)	0.0237 (3)	
H40A	0.666174	0.245312	0.695612	0.028*	
H40B	0.662338	0.295150	0.577397	0.028*	
C41	0.57382 (9)	0.25823 (8)	0.62754 (19)	0.0233 (3)	
H41A	0.554484	0.239663	0.707006	0.028*	
H41B	0.575640	0.224634	0.559928	0.028*	
C42	0.53237 (8)	0.31323 (7)	0.57869 (17)	0.0194 (3)	
H42A	0.549182	0.328324	0.493887	0.023*	
H42B	0.488620	0.297426	0.563883	0.023*	
C43	0.42730 (7)	0.42635 (7)	0.69037 (16)	0.0168 (2)	
H43	0.403459	0.460602	0.643590	0.020*	
C44	0.38717 (8)	0.36572 (8)	0.67626 (18)	0.0211 (3)	
H44A	0.409026	0.330265	0.721293	0.025*	
H44B	0.383867	0.354579	0.582577	0.025*	
C45	0.31995 (8)	0.37302 (10)	0.73308 (19)	0.0266 (3)	
H45A	0.295738	0.403788	0.679399	0.032*	
H45B	0.297744	0.331577	0.728571	0.032*	
C46	0.32096 (9)	0.39577 (10)	0.87375 (19)	0.0253 (3)	
H46A	0.338704	0.361896	0.930138	0.030*	
H46B	0.276953	0.404482	0.902988	0.030*	
C47	0.36079 (8)	0.45570 (9)	0.88819 (19)	0.0244 (3)	
H47A	0.363015	0.467713	0.981542	0.029*	
H47B	0.340163	0.490970	0.840199	0.029*	
C48	0.42844 (8)	0.44587 (8)	0.83523 (16)	0.0203 (3)	
H48A	0.450040	0.412428	0.886828	0.024*	
H48B	0.452947	0.485771	0.845052	0.024*	

### Atomic displacement parameters (Å^2^)

**Table:**

	*U*^11^	*U*^22^	*U*^33^	*U*^12^	*U*^13^	*U*^23^
Zr1	0.00815 (7)	0.01012 (8)	0.00874 (6)	−0.00008 (6)	0.000	0.000
N1	0.0123 (4)	0.0157 (5)	0.0125 (4)	0.0019 (4)	−0.0011 (4)	0.0002 (4)
N2	0.0118 (4)	0.0142 (5)	0.0137 (5)	−0.0003 (4)	−0.0008 (4)	−0.0020 (4)
C1	0.0111 (5)	0.0170 (5)	0.0120 (5)	0.0009 (4)	−0.0015 (4)	0.0005 (4)
C2	0.0138 (5)	0.0207 (6)	0.0144 (5)	−0.0005 (5)	0.0003 (5)	−0.0002 (5)
C3	0.0145 (6)	0.0245 (7)	0.0196 (6)	0.0006 (5)	−0.0016 (5)	−0.0062 (5)
C4	0.0141 (6)	0.0334 (8)	0.0194 (7)	−0.0025 (5)	−0.0041 (5)	−0.0048 (6)
C5	0.0137 (6)	0.0308 (8)	0.0205 (7)	−0.0053 (5)	−0.0012 (5)	−0.0016 (6)
C6	0.0122 (5)	0.0261 (7)	0.0155 (6)	−0.0005 (5)	0.0003 (5)	−0.0007 (5)
C7	0.0167 (6)	0.0176 (6)	0.0148 (6)	0.0042 (5)	−0.0003 (5)	0.0003 (5)
C8	0.0184 (6)	0.0239 (7)	0.0187 (6)	0.0075 (5)	0.0002 (5)	−0.0021 (5)
C9	0.0356 (10)	0.0307 (9)	0.0282 (9)	0.0200 (8)	−0.0068 (8)	−0.0043 (7)
C10	0.0607 (15)	0.0201 (8)	0.0271 (9)	0.0162 (9)	0.0058 (9)	0.0028 (6)
C11	0.0532 (13)	0.0188 (7)	0.0243 (8)	0.0049 (8)	0.0107 (9)	0.0047 (6)
C12	0.0258 (7)	0.0151 (6)	0.0205 (7)	0.0020 (5)	0.0085 (6)	0.0005 (5)
C13	0.0130 (5)	0.0136 (5)	0.0135 (5)	0.0004 (4)	−0.0010 (4)	−0.0011 (4)
C14	0.0189 (6)	0.0143 (6)	0.0156 (6)	0.0013 (4)	0.0004 (5)	−0.0004 (4)
C15	0.0205 (7)	0.0169 (6)	0.0188 (6)	0.0045 (5)	−0.0001 (5)	−0.0017 (5)
C16	0.0250 (7)	0.0169 (6)	0.0201 (7)	0.0021 (5)	0.0040 (6)	−0.0033 (5)
C17	0.0204 (6)	0.0166 (6)	0.0200 (7)	0.0009 (5)	0.0058 (5)	−0.0004 (5)
C18	0.0172 (6)	0.0169 (6)	0.0148 (6)	0.0010 (5)	0.0008 (4)	0.0004 (4)
C19	0.0130 (5)	0.0144 (5)	0.0146 (5)	−0.0015 (4)	−0.0002 (4)	−0.0005 (4)
C20	0.0153 (6)	0.0156 (6)	0.0186 (6)	−0.0022 (4)	−0.0002 (5)	0.0020 (5)
C21	0.0175 (6)	0.0189 (6)	0.0232 (7)	−0.0053 (5)	−0.0002 (5)	−0.0007 (5)
C22	0.0137 (6)	0.0256 (7)	0.0208 (7)	−0.0032 (5)	−0.0009 (5)	−0.0013 (6)
C23	0.0145 (6)	0.0238 (7)	0.0187 (6)	−0.0011 (5)	−0.0032 (5)	0.0015 (5)
C24	0.0127 (5)	0.0170 (6)	0.0197 (6)	−0.0004 (4)	−0.0016 (5)	0.0023 (5)
Zr2	0.00851 (6)	0.00851 (6)	0.00787 (10)	0.000	0.000	0.000
N3	0.0097 (4)	0.0107 (4)	0.0124 (5)	−0.0004 (3)	0.0007 (4)	0.0017 (4)
C25	0.0125 (5)	0.0089 (5)	0.0116 (5)	0.0001 (4)	0.0009 (4)	0.0004 (4)
C26	0.0164 (5)	0.0107 (5)	0.0122 (5)	0.0001 (4)	−0.0007 (4)	−0.0001 (4)
C27	0.0208 (6)	0.0139 (5)	0.0173 (6)	0.0003 (5)	−0.0063 (5)	0.0011 (5)
C28	0.0268 (7)	0.0128 (5)	0.0182 (6)	0.0022 (5)	−0.0060 (5)	0.0026 (5)
C29	0.0194 (6)	0.0125 (5)	0.0192 (6)	0.0032 (5)	−0.0019 (5)	0.0002 (5)
C30	0.0154 (5)	0.0101 (5)	0.0152 (5)	−0.0002 (4)	−0.0003 (4)	−0.0001 (4)
C31	0.0114 (5)	0.0118 (5)	0.0135 (5)	−0.0022 (4)	−0.0002 (4)	−0.0011 (4)
C32	0.0145 (5)	0.0136 (5)	0.0154 (6)	−0.0024 (4)	0.0010 (4)	−0.0019 (4)
C33	0.0158 (6)	0.0155 (6)	0.0249 (7)	−0.0045 (4)	0.0002 (5)	−0.0006 (5)
C34	0.0133 (6)	0.0227 (7)	0.0203 (7)	−0.0029 (5)	0.0007 (5)	0.0007 (5)
C35	0.0143 (6)	0.0228 (7)	0.0170 (6)	0.0001 (5)	0.0031 (5)	−0.0015 (5)
C36	0.0152 (5)	0.0146 (5)	0.0152 (6)	0.0003 (4)	0.0019 (4)	−0.0018 (4)
Zr3	0.01181 (7)	0.01181 (7)	0.01390 (12)	0.000	0.000	0.000
N4	0.0132 (5)	0.0144 (5)	0.0170 (5)	0.0013 (4)	0.0008 (4)	0.0030 (4)
C37	0.0163 (6)	0.0166 (6)	0.0170 (6)	0.0008 (4)	−0.0007 (5)	0.0009 (5)
C38	0.0180 (6)	0.0190 (6)	0.0237 (7)	0.0016 (5)	−0.0041 (5)	−0.0027 (5)
C39	0.0211 (7)	0.0252 (7)	0.0244 (7)	0.0050 (6)	−0.0062 (6)	−0.0023 (6)
C40	0.0221 (7)	0.0220 (7)	0.0269 (8)	0.0058 (6)	−0.0012 (6)	−0.0002 (6)
C41	0.0268 (8)	0.0160 (6)	0.0271 (8)	0.0026 (5)	−0.0026 (6)	0.0000 (6)
C42	0.0215 (7)	0.0151 (6)	0.0214 (7)	0.0003 (5)	−0.0037 (5)	−0.0003 (5)
C43	0.0152 (5)	0.0189 (6)	0.0162 (5)	0.0002 (4)	0.0001 (5)	0.0033 (5)
C44	0.0180 (6)	0.0231 (7)	0.0222 (7)	−0.0040 (5)	0.0010 (5)	0.0023 (6)
C45	0.0182 (7)	0.0359 (9)	0.0258 (8)	−0.0066 (6)	0.0015 (6)	0.0037 (7)
C46	0.0205 (7)	0.0312 (8)	0.0242 (8)	−0.0012 (6)	0.0029 (6)	0.0075 (7)
C47	0.0203 (7)	0.0286 (8)	0.0245 (7)	0.0019 (6)	0.0054 (6)	0.0004 (6)
C48	0.0180 (6)	0.0240 (7)	0.0188 (7)	0.0009 (5)	0.0012 (5)	0.0003 (5)

### Geometric parameters (Å, º)

**Table:**

Zr1—N1^i^	2.0908 (12)	Zr2—N3^iv^	2.0973 (12)
Zr1—N1	2.0908 (12)	Zr2—N3	2.0973 (12)
Zr1—N2	2.0928 (12)	N3—C31	1.4756 (18)
Zr1—N2^i^	2.0929 (12)	N3—C25	1.4863 (18)
N1—C7	1.4816 (19)	C25—C26	1.541 (2)
N1—C1	1.4821 (19)	C25—C30	1.5441 (18)
N2—C19	1.4790 (18)	C25—H25	1.0000
N2—C13	1.4854 (19)	C26—C27	1.534 (2)
C1—C6	1.536 (2)	C26—H26A	0.9900
C1—C2	1.542 (2)	C26—H26B	0.9900
C1—H1	1.0000	C27—C28	1.527 (2)
C2—C3	1.530 (2)	C27—H27A	0.9900
C2—H2A	0.9900	C27—H27B	0.9900
C2—H2B	0.9900	C28—C29	1.523 (2)
C3—C4	1.520 (2)	C28—H28A	0.9900
C3—H3A	0.9900	C28—H28B	0.9900
C3—H3B	0.9900	C29—C30	1.535 (2)
C4—C5	1.531 (3)	C29—H29A	0.9900
C4—H4A	0.9900	C29—H29B	0.9900
C4—H4B	0.9900	C30—H30A	0.9900
C5—C6	1.530 (2)	C30—H30B	0.9900
C5—H5A	0.9900	C31—C32	1.531 (2)
C5—H5B	0.9900	C31—C36	1.531 (2)
C6—H6A	0.9900	C31—H31	1.0000
C6—H6B	0.9900	C32—C33	1.536 (2)
C7—C12	1.523 (2)	C32—H32A	0.9900
C7—C8	1.532 (2)	C32—H32B	0.9900
C7—H7	1.0000	C33—C34	1.527 (2)
C8—C9	1.526 (3)	C33—H33A	0.9900
C8—H8A	0.9900	C33—H33B	0.9900
C8—H8B	0.9900	C34—C35	1.527 (2)
C9—C10	1.535 (4)	C34—H34A	0.9900
C9—H9A	0.9900	C34—H34B	0.9900
C9—H9B	0.9900	C35—C36	1.531 (2)
C10—C11	1.519 (3)	C35—H35A	0.9900
C10—H10A	0.9900	C35—H35B	0.9900
C10—H10B	0.9900	C36—H36A	0.9900
C11—C12	1.532 (2)	C36—H36B	0.9900
C11—H11A	0.9900	Zr3—N4^v^	2.0940 (13)
C11—H11B	0.9900	Zr3—N4^vi^	2.0940 (13)
C12—H12A	0.9900	Zr3—N4^vii^	2.0940 (13)
C12—H12B	0.9900	Zr3—N4	2.0940 (13)
C13—C14	1.536 (2)	N4—C37	1.473 (2)
C13—C18	1.538 (2)	N4—C43	1.488 (2)
C13—H13	1.0000	C37—C38	1.526 (2)
C14—C15	1.537 (2)	C37—C42	1.532 (2)
C14—H14A	0.9900	C37—H37	1.0000
C14—H14B	0.9900	C38—C39	1.534 (2)
C15—C16	1.525 (2)	C38—H38A	0.9900
C15—H15A	0.9900	C38—H38B	0.9900
C15—H15B	0.9900	C39—C40	1.527 (3)
C16—C17	1.524 (2)	C39—H39A	0.9900
C16—H16A	0.9900	C39—H39B	0.9900
C16—H16B	0.9900	C40—C41	1.528 (3)
C17—C18	1.534 (2)	C40—H40A	0.9900
C17—H17A	0.9900	C40—H40B	0.9900
C17—H17B	0.9900	C41—C42	1.532 (2)
C18—H18A	0.9900	C41—H41A	0.9900
C18—H18B	0.9900	C41—H41B	0.9900
C19—C20	1.533 (2)	C42—H42A	0.9900
C19—C24	1.534 (2)	C42—H42B	0.9900
C19—H19	1.0000	C43—C44	1.536 (2)
C20—C21	1.532 (2)	C43—C48	1.539 (2)
C20—H20A	0.9900	C43—H43	1.0000
C20—H20B	0.9900	C44—C45	1.537 (2)
C21—C22	1.523 (2)	C44—H44A	0.9900
C21—H21A	0.9900	C44—H44B	0.9900
C21—H21B	0.9900	C45—C46	1.518 (3)
C22—C23	1.525 (2)	C45—H45A	0.9900
C22—H22A	0.9900	C45—H45B	0.9900
C22—H22B	0.9900	C46—C47	1.521 (3)
C23—C24	1.530 (2)	C46—H46A	0.9900
C23—H23A	0.9900	C46—H46B	0.9900
C23—H23B	0.9900	C47—C48	1.537 (2)
C24—H24A	0.9900	C47—H47A	0.9900
C24—H24B	0.9900	C47—H47B	0.9900
Zr2—N3^ii^	2.0973 (12)	C48—H48A	0.9900
Zr2—N3^iii^	2.0973 (12)	C48—H48B	0.9900
			
N1^i^—Zr1—N1	104.75 (7)	N3^ii^—Zr2—N3	104.85 (7)
N1^i^—Zr1—N2	112.22 (5)	N3^iii^—Zr2—N3	111.83 (4)
N1—Zr1—N2	111.14 (5)	N3^iv^—Zr2—N3	111.83 (4)
N1^i^—Zr1—N2^i^	111.14 (5)	C31—N3—C25	113.79 (11)
N1—Zr1—N2^i^	112.22 (5)	C31—N3—Zr2	136.41 (9)
N2—Zr1—N2^i^	105.53 (7)	C25—N3—Zr2	109.37 (8)
C7—N1—C1	113.64 (11)	N3—C25—C26	115.01 (11)
C7—N1—Zr1	135.02 (10)	N3—C25—C30	112.72 (11)
C1—N1—Zr1	110.57 (9)	C26—C25—C30	109.02 (11)
C19—N2—C13	113.77 (11)	N3—C25—H25	106.5
C19—N2—Zr1	135.54 (9)	C26—C25—H25	106.5
C13—N2—Zr1	110.12 (8)	C30—C25—H25	106.5
N1—C1—C6	113.38 (12)	C27—C26—C25	111.53 (12)
N1—C1—C2	114.84 (12)	C27—C26—H26A	109.3
C6—C1—C2	108.99 (12)	C25—C26—H26A	109.3
N1—C1—H1	106.3	C27—C26—H26B	109.3
C6—C1—H1	106.3	C25—C26—H26B	109.3
C2—C1—H1	106.3	H26A—C26—H26B	108.0
C3—C2—C1	111.77 (13)	C28—C27—C26	111.46 (12)
C3—C2—H2A	109.3	C28—C27—H27A	109.3
C1—C2—H2A	109.3	C26—C27—H27A	109.3
C3—C2—H2B	109.3	C28—C27—H27B	109.3
C1—C2—H2B	109.3	C26—C27—H27B	109.3
H2A—C2—H2B	107.9	H27A—C27—H27B	108.0
C4—C3—C2	111.63 (13)	C29—C28—C27	110.63 (13)
C4—C3—H3A	109.3	C29—C28—H28A	109.5
C2—C3—H3A	109.3	C27—C28—H28A	109.5
C4—C3—H3B	109.3	C29—C28—H28B	109.5
C2—C3—H3B	109.3	C27—C28—H28B	109.5
H3A—C3—H3B	108.0	H28A—C28—H28B	108.1
C3—C4—C5	110.50 (13)	C28—C29—C30	111.40 (13)
C3—C4—H4A	109.5	C28—C29—H29A	109.3
C5—C4—H4A	109.5	C30—C29—H29A	109.3
C3—C4—H4B	109.5	C28—C29—H29B	109.3
C5—C4—H4B	109.5	C30—C29—H29B	109.3
H4A—C4—H4B	108.1	H29A—C29—H29B	108.0
C6—C5—C4	110.62 (14)	C29—C30—C25	113.05 (11)
C6—C5—H5A	109.5	C29—C30—H30A	109.0
C4—C5—H5A	109.5	C25—C30—H30A	109.0
C6—C5—H5B	109.5	C29—C30—H30B	109.0
C4—C5—H5B	109.5	C25—C30—H30B	109.0
H5A—C5—H5B	108.1	H30A—C30—H30B	107.8
C5—C6—C1	113.08 (13)	N3—C31—C32	112.82 (11)
C5—C6—H6A	109.0	N3—C31—C36	111.90 (11)
C1—C6—H6A	109.0	C32—C31—C36	110.75 (11)
C5—C6—H6B	109.0	N3—C31—H31	107.0
C1—C6—H6B	109.0	C32—C31—H31	107.0
H6A—C6—H6B	107.8	C36—C31—H31	107.0
N1—C7—C12	110.60 (12)	C31—C32—C33	112.51 (12)
N1—C7—C8	112.79 (13)	C31—C32—H32A	109.1
C12—C7—C8	111.04 (13)	C33—C32—H32A	109.1
N1—C7—H7	107.4	C31—C32—H32B	109.1
C12—C7—H7	107.4	C33—C32—H32B	109.1
C8—C7—H7	107.4	H32A—C32—H32B	107.8
C9—C8—C7	112.20 (15)	C34—C33—C32	111.26 (12)
C9—C8—H8A	109.2	C34—C33—H33A	109.4
C7—C8—H8A	109.2	C32—C33—H33A	109.4
C9—C8—H8B	109.2	C34—C33—H33B	109.4
C7—C8—H8B	109.2	C32—C33—H33B	109.4
H8A—C8—H8B	107.9	H33A—C33—H33B	108.0
C8—C9—C10	110.91 (16)	C35—C34—C33	109.93 (13)
C8—C9—H9A	109.5	C35—C34—H34A	109.7
C10—C9—H9A	109.5	C33—C34—H34A	109.7
C8—C9—H9B	109.5	C35—C34—H34B	109.7
C10—C9—H9B	109.5	C33—C34—H34B	109.7
H9A—C9—H9B	108.0	H34A—C34—H34B	108.2
C11—C10—C9	110.67 (18)	C34—C35—C36	112.09 (13)
C11—C10—H10A	109.5	C34—C35—H35A	109.2
C9—C10—H10A	109.5	C36—C35—H35A	109.2
C11—C10—H10B	109.5	C34—C35—H35B	109.2
C9—C10—H10B	109.5	C36—C35—H35B	109.2
H10A—C10—H10B	108.1	H35A—C35—H35B	107.9
C10—C11—C12	111.84 (16)	C31—C36—C35	111.88 (12)
C10—C11—H11A	109.2	C31—C36—H36A	109.2
C12—C11—H11A	109.2	C35—C36—H36A	109.2
C10—C11—H11B	109.2	C31—C36—H36B	109.2
C12—C11—H11B	109.2	C35—C36—H36B	109.2
H11A—C11—H11B	107.9	H36A—C36—H36B	107.9
C7—C12—C11	112.70 (16)	N4^v^—Zr3—N4^vi^	112.00 (4)
C7—C12—H12A	109.1	N4^v^—Zr3—N4^vii^	112.00 (4)
C11—C12—H12A	109.1	N4^vi^—Zr3—N4^vii^	104.53 (8)
C7—C12—H12B	109.1	N4^v^—Zr3—N4	104.53 (8)
C11—C12—H12B	109.1	N4^vi^—Zr3—N4	112.00 (4)
H12A—C12—H12B	107.8	N4^vii^—Zr3—N4	112.00 (4)
N2—C13—C14	113.60 (12)	C37—N4—C43	113.49 (12)
N2—C13—C18	115.19 (12)	C37—N4—Zr3	138.00 (10)
C14—C13—C18	108.70 (12)	C43—N4—Zr3	108.15 (9)
N2—C13—H13	106.2	N4—C37—C38	112.30 (13)
C14—C13—H13	106.2	N4—C37—C42	112.25 (13)
C18—C13—H13	106.2	C38—C37—C42	110.07 (13)
C13—C14—C15	112.93 (13)	N4—C37—H37	107.3
C13—C14—H14A	109.0	C38—C37—H37	107.3
C15—C14—H14A	109.0	C42—C37—H37	107.3
C13—C14—H14B	109.0	C37—C38—C39	112.30 (14)
C15—C14—H14B	109.0	C37—C38—H38A	109.1
H14A—C14—H14B	107.8	C39—C38—H38A	109.1
C16—C15—C14	111.48 (13)	C37—C38—H38B	109.1
C16—C15—H15A	109.3	C39—C38—H38B	109.1
C14—C15—H15A	109.3	H38A—C38—H38B	107.9
C16—C15—H15B	109.3	C40—C39—C38	112.12 (14)
C14—C15—H15B	109.3	C40—C39—H39A	109.2
H15A—C15—H15B	108.0	C38—C39—H39A	109.2
C17—C16—C15	111.16 (13)	C40—C39—H39B	109.2
C17—C16—H16A	109.4	C38—C39—H39B	109.2
C15—C16—H16A	109.4	H39A—C39—H39B	107.9
C17—C16—H16B	109.4	C39—C40—C41	110.09 (14)
C15—C16—H16B	109.4	C39—C40—H40A	109.6
H16A—C16—H16B	108.0	C41—C40—H40A	109.6
C16—C17—C18	111.54 (13)	C39—C40—H40B	109.6
C16—C17—H17A	109.3	C41—C40—H40B	109.6
C18—C17—H17A	109.3	H40A—C40—H40B	108.2
C16—C17—H17B	109.3	C40—C41—C42	111.09 (14)
C18—C17—H17B	109.3	C40—C41—H41A	109.4
H17A—C17—H17B	108.0	C42—C41—H41A	109.4
C17—C18—C13	111.56 (13)	C40—C41—H41B	109.4
C17—C18—H18A	109.3	C42—C41—H41B	109.4
C13—C18—H18A	109.3	H41A—C41—H41B	108.0
C17—C18—H18B	109.3	C37—C42—C41	113.01 (14)
C13—C18—H18B	109.3	C37—C42—H42A	109.0
H18A—C18—H18B	108.0	C41—C42—H42A	109.0
N2—C19—C20	112.63 (12)	C37—C42—H42B	109.0
N2—C19—C24	111.22 (11)	C41—C42—H42B	109.0
C20—C19—C24	110.52 (12)	H42A—C42—H42B	107.8
N2—C19—H19	107.4	N4—C43—C44	113.25 (13)
C20—C19—H19	107.4	N4—C43—C48	115.83 (13)
C24—C19—H19	107.4	C44—C43—C48	108.70 (13)
C21—C20—C19	112.44 (13)	N4—C43—H43	106.1
C21—C20—H20A	109.1	C44—C43—H43	106.1
C19—C20—H20A	109.1	C48—C43—H43	106.1
C21—C20—H20B	109.1	C43—C44—C45	112.76 (15)
C19—C20—H20B	109.1	C43—C44—H44A	109.0
H20A—C20—H20B	107.8	C45—C44—H44A	109.0
C22—C21—C20	111.40 (13)	C43—C44—H44B	109.0
C22—C21—H21A	109.3	C45—C44—H44B	109.0
C20—C21—H21A	109.3	H44A—C44—H44B	107.8
C22—C21—H21B	109.3	C46—C45—C44	112.21 (15)
C20—C21—H21B	109.3	C46—C45—H45A	109.2
H21A—C21—H21B	108.0	C44—C45—H45A	109.2
C21—C22—C23	110.12 (13)	C46—C45—H45B	109.2
C21—C22—H22A	109.6	C44—C45—H45B	109.2
C23—C22—H22A	109.6	H45A—C45—H45B	107.9
C21—C22—H22B	109.6	C45—C46—C47	111.17 (15)
C23—C22—H22B	109.6	C45—C46—H46A	109.4
H22A—C22—H22B	108.2	C47—C46—H46A	109.4
C22—C23—C24	111.62 (13)	C45—C46—H46B	109.4
C22—C23—H23A	109.3	C47—C46—H46B	109.4
C24—C23—H23A	109.3	H46A—C46—H46B	108.0
C22—C23—H23B	109.3	C46—C47—C48	111.36 (15)
C24—C23—H23B	109.3	C46—C47—H47A	109.4
H23A—C23—H23B	108.0	C48—C47—H47A	109.4
C23—C24—C19	111.76 (13)	C46—C47—H47B	109.4
C23—C24—H24A	109.3	C48—C47—H47B	109.4
C19—C24—H24A	109.3	H47A—C47—H47B	108.0
C23—C24—H24B	109.3	C47—C48—C43	111.19 (14)
C19—C24—H24B	109.3	C47—C48—H48A	109.4
H24A—C24—H24B	107.9	C43—C48—H48A	109.4
N3^ii^—Zr2—N3^iii^	111.83 (4)	C47—C48—H48B	109.4
N3^ii^—Zr2—N3^iv^	111.83 (4)	C43—C48—H48B	109.4
N3^iii^—Zr2—N3^iv^	104.85 (7)	H48A—C48—H48B	108.0
			
C7—N1—C1—C6	−60.51 (16)	C31—N3—C25—C26	66.47 (15)
Zr1—N1—C1—C6	128.03 (11)	Zr2—N3—C25—C26	−107.23 (11)
C7—N1—C1—C2	65.70 (15)	C31—N3—C25—C30	−59.35 (15)
Zr1—N1—C1—C2	−105.76 (11)	Zr2—N3—C25—C30	126.95 (10)
N1—C1—C2—C3	177.00 (12)	N3—C25—C26—C27	177.03 (11)
C6—C1—C2—C3	−54.55 (16)	C30—C25—C26—C27	−55.26 (14)
C1—C2—C3—C4	56.96 (18)	C25—C26—C27—C28	57.80 (17)
C2—C3—C4—C5	−56.6 (2)	C26—C27—C28—C29	−56.56 (18)
C3—C4—C5—C6	55.6 (2)	C27—C28—C29—C30	54.61 (18)
C4—C5—C6—C1	−56.12 (19)	C28—C29—C30—C25	−54.78 (17)
N1—C1—C6—C5	−175.90 (13)	N3—C25—C30—C29	−176.80 (12)
C2—C1—C6—C5	54.84 (17)	C26—C25—C30—C29	54.21 (15)
C1—N1—C7—C12	−134.13 (13)	C25—N3—C31—C32	102.30 (13)
Zr1—N1—C7—C12	34.53 (19)	Zr2—N3—C31—C32	−86.34 (15)
C1—N1—C7—C8	100.85 (15)	C25—N3—C31—C36	−132.02 (12)
Zr1—N1—C7—C8	−90.50 (16)	Zr2—N3—C31—C36	39.35 (18)
N1—C7—C8—C9	178.24 (14)	N3—C31—C32—C33	179.41 (12)
C12—C7—C8—C9	53.45 (19)	C36—C31—C32—C33	53.10 (16)
C7—C8—C9—C10	−56.0 (2)	C31—C32—C33—C34	−55.50 (18)
C8—C9—C10—C11	56.4 (2)	C32—C33—C34—C35	56.09 (18)
C9—C10—C11—C12	−55.2 (3)	C33—C34—C35—C36	−56.52 (18)
N1—C7—C12—C11	−177.98 (14)	N3—C31—C36—C35	−179.52 (12)
C8—C7—C12—C11	−51.97 (19)	C32—C31—C36—C35	−52.70 (16)
C10—C11—C12—C7	53.7 (2)	C34—C35—C36—C31	55.46 (17)
C19—N2—C13—C14	59.28 (16)	C43—N4—C37—C38	−130.74 (14)
Zr1—N2—C13—C14	−127.95 (10)	Zr3—N4—C37—C38	41.3 (2)
C19—N2—C13—C18	−67.03 (15)	C43—N4—C37—C42	104.61 (15)
Zr1—N2—C13—C18	105.75 (12)	Zr3—N4—C37—C42	−83.35 (18)
N2—C13—C14—C15	174.94 (12)	N4—C37—C38—C39	−178.72 (14)
C18—C13—C14—C15	−55.41 (16)	C42—C37—C38—C39	−52.88 (18)
C13—C14—C15—C16	54.66 (18)	C37—C38—C39—C40	55.3 (2)
C14—C15—C16—C17	−53.20 (19)	C38—C39—C40—C41	−55.7 (2)
C15—C16—C17—C18	55.06 (19)	C39—C40—C41—C42	55.5 (2)
C16—C17—C18—C13	−57.60 (18)	N4—C37—C42—C41	179.54 (13)
N2—C13—C18—C17	−174.76 (12)	C38—C37—C42—C41	53.66 (18)
C14—C13—C18—C17	56.47 (16)	C40—C41—C42—C37	−55.8 (2)
C13—N2—C19—C20	−103.47 (14)	C37—N4—C43—C44	−58.69 (18)
Zr1—N2—C19—C20	86.23 (16)	Zr3—N4—C43—C44	126.91 (12)
C13—N2—C19—C24	131.82 (13)	C37—N4—C43—C48	67.88 (17)
Zr1—N2—C19—C24	−38.48 (19)	Zr3—N4—C43—C48	−106.52 (13)
N2—C19—C20—C21	−178.17 (12)	N4—C43—C44—C45	−174.77 (14)
C24—C19—C20—C21	−53.08 (17)	C48—C43—C44—C45	54.97 (18)
C19—C20—C21—C22	55.22 (18)	C43—C44—C45—C46	−53.7 (2)
C20—C21—C22—C23	−56.17 (18)	C44—C45—C46—C47	52.7 (2)
C21—C22—C23—C24	56.95 (18)	C45—C46—C47—C48	−55.2 (2)
C22—C23—C24—C19	−56.19 (18)	C46—C47—C48—C43	58.40 (19)
N2—C19—C24—C23	179.23 (12)	N4—C43—C48—C47	174.08 (13)
C20—C19—C24—C23	53.35 (17)	C44—C43—C48—C47	−57.09 (17)

Symmetry codes: (i) −*x*+1, −*y*, *z*; (ii) −*x*, −*y*, *z*; (iii) *y*, −*x*, −*z*+1; (iv) −*y*, *x*, −*z*+1; (v) −*x*+1, −*y*+1, *z*; (vi) *y*, −*x*+1, −*z*+1; (vii) −*y*+1, *x*, −*z*+1.

## References

[bb1] Adler, C., Bekurdts, A., Haase, D., Saak, W., Schmidtmann, M. & Beckhaus, R. (2014*b*). *Eur. J. Inorg. Chem.* pp. 1289–1302.

[bb2] Adler, C., Tomaschun, G., Schmidtmann, M. & Beckhaus, R. (2014*a*). *Organometallics*, **33**, 7011–7014.

[bb3] Brandenburg, K. & Putz, H. (2006). *DIAMOND*. Crystal Impact GbR, Bonn, Germany.

[bb4] Bruker (2015). *APEX3* and *SAINT*. Bruker AXS Inc., Madison, Wisconsin, USA.

[bb5] Diamond, G. M., Jordan, R. F. & Petersen, J. L. (1996). *Organometallics*, **15**, 4030–4037.

[bb6] Diamond, G. M., Rodewald, S. & Jordan, R. F. (1995). *Organometallics*, **14**, 5–7.

[bb7] Duan, Z., Thomas, L. M. & Verkade, J. G. (1997). *Polyhedron*, **16**, 635–641.

[bb8] Guérin, F., Stewart, J. C., Beddie, C. & Stephan, D. W. (2000). *Organometallics*, **19**, 2994–3000.

[bb9] Hitchcock, P. B., Lappert, M. F. & Protchenko, A. V. (2006). *Chem. Commun.* pp. 3546–3548. 10.1039/b607429d 16921440

[bb10] Kasani, A., Gambarotta, S. & Bensimon, C. (1997). *Can. J. Chem.* **75**, 1494–1499.

[bb11] Kempe, R. (2000). *Angew. Chem. Int. Ed.* **39**, 468–493. 10.1002/(sici)1521-3773(20000204)39:3<468::aid-anie468>3.0.co;2-g 10671235

[bb12] Krause, L., Herbst-Irmer, R., Sheldrick, G. M. & Stalke, D. (2015). *J. Appl. Cryst.* **48**, 3–10. 10.1107/S1600576714022985 PMC4453166 26089746

[bb13] Motolko, K. S. A., Price, J. S., Emslie, D. J. H., Jenkins, H. A. & Britten, J. F. (2017). *Organometallics*, **36**, 3084–3093.

[bb14] Pyykkö, P. & Atsumi, M. (2009). *Chem. Eur. J.* **15**, 12770–12779. 10.1002/chem.200901472 19856342

[bb15] Roesky, P. W. (2009). *Angew. Chem. Int. Ed.* **48**, 4892–4894. 10.1002/anie.200900735 19437521

[bb16] Scherer, W., Wolstenholme, D. J., Herz, V., Eickerling, G., Brück, A., Benndorf, P. & Roesky, P. W. (2010). *Angew. Chem. Int. Ed.* **49**, 2242–2246. 10.1002/anie.200905463 20187050

[bb17] Shafir, A. & Arnold, A. (2001). *J. Am. Chem. Soc.* **123**, 9212–9213. 10.1021/ja0161857 11552850

[bb18] Sheldrick, G. M. (2008). *Acta Cryst.* A**64**, 112–122. 10.1107/S0108767307043930 18156677

[bb19] Sheldrick, G. M. (2015). *Acta Cryst.* C**71**, 3–8.

[bb20] Westrip, S. P. (2010). *J. Appl. Cryst.* **43**, 920–925.

[bb21] Yu, X., Bi, S., Guzei, I. A., Lin, Z. & Xue, Z.-L. (2004). *Inorg. Chem.* **43**, 7111–7119. 10.1021/ic049023v 15500349

